# Effectiveness and Safety of Personalized Cholic Acid Treatment in Patients With Bile Acid Synthesis Defects

**DOI:** 10.1002/jimd.70062

**Published:** 2025-07-11

**Authors:** Yasmin Polak, Elles Marleen Kemper, Marc Engelen, Femke C. C. Klouwer, Kevin Berendse, Frédéric M. Vaz, Bart G. P. Koot, Eleonora (Noortje) L. Swart, Carla E. M. Hollak

**Affiliations:** ^1^ Platform Medicine for Society Amsterdam the Netherlands; ^2^ Department of Pharmacy and Clinical Pharmacology Amsterdam UMC, University of Amsterdam Amsterdam the Netherlands; ^3^ Department of Pediatric Neurology Amsterdam UMC, University of Amsterdam Amsterdam the Netherlands; ^4^ The Pediatric Practice (de Kinderartsenpraktijk) Amsterdam the Netherlands; ^5^ Department of Laboratory Medicine and Pediatrics Laboratory Genetic Metabolic Diseases, Emma Children's Hospital, Amsterdam UMC, University of Amsterdam Amsterdam the Netherlands; ^6^ Department of Pediatric Gastroenterology Emma Children's Hospital, Amsterdam UMC, University of Amsterdam Amsterdam the Netherlands; ^7^ Department of Endocrinology and Metabolism Amsterdam UMC, University of Amsterdam Amsterdam the Netherlands

**Keywords:** 3β‐hydroxy‐Δ^5^‐C_27_‐steroid oxidoreductase (3β‐HSD), bile acid synthesis, cholic acid, peroxisomal disorder, single enzyme defects, toxic bile acid intermediates, α‐methylacyl‐CoA racemase (AMACR)

## Abstract

Bile acid synthesis defects (BASDs) comprise a group of rare, often severe, metabolic disorders. Bile acid replacement therapy decreases toxic bile acid intermediates production and improves biochemical profiles, potentially delaying or stabilizing disease progression. An open label, non‐randomized trial with cholic acid (CA) supplementation included six patients with α‐methylacyl‐CoA racemase (AMACR) deficiency and one patient with 3β‐hydroxy‐Δ^5^‐C_27_‐steroid oxidoreductase deficiency. Patients received up to 20 mg/kg/day CA for 3.5 years, adjusted for biochemical response, side effects, and clinical evaluation. Bile acid metabolites, liver enzymes, liver stiffness, and neurological symptoms were evaluated at baseline and during follow‐up. CA was well tolerated in children (*n* = 3), allowing for higher doses. Adults (*n* = 4) experienced more side effects, primarily diarrhea and other gastrointestinal symptoms. Children's transaminase levels normalized during treatment, while adults' levels remained normal throughout. Elevated C_27_‐bile acid intermediates, C_29_‐dicarboxylic acid, and pristanic acid were observed in all AMACR patients. C_27_‐bile acids and C_29_‐dicarboxylic acid decreased with treatment, while pristanic acid fluctuated and remained elevated. No clinically relevant changes were observed in liver elasticity, fat‐soluble vitamin levels, neurological assessment, or growth (in children). One adult developed hepatocellular carcinoma during treatment. CA treatment is generally safe, with acceptable tolerance and a marked biochemical response observed in children, although biomarker levels remained markedly elevated. In adults, however, the balance shifts negatively, with side effects outweighing the (biochemical) benefits. A longer study is necessary to evaluate the impact of CA treatment on the clinical relevance of the observed biochemical response.

## 1 Introduction

Bile acid synthesis defects (BASDs) are a group of rare metabolic disorders characterized by defects in the synthesis and metabolism of bile acids. These disorders can be severely disabling or even fatal [1, 2, 3, 4]. BASDs include two main categories: single enzyme defects (SEDs) in bile acid synthesis and generalized defects such as those caused by peroxisomal disorders (e.g., mutations in PEX genes as seen in Zellweger spectrum disorder [ZSD]) that affect multiple metabolic pathways, including bile acid synthesis [5, 6]. SEDs result from inherited deficiencies in enzymes catalyzing key reactions in the production of primary bile acids cholic acid (CA) and chenodeoxycholic acid (CDCA) [2, 4]. Bile acids perform essential physiological roles, including promoting bile flow and excretion, aiding in the intestinal absorption of fats and fat‐soluble vitamins, and serving as the primary pathway for cholesterol catabolism and elimination via conversion into bile acids [2, 4, 7, 8, 9]. In certain BASDs involving the peroxisomal steps, toxic C_27_‐bile acid intermediates, such as dihydroxycholestanoic acid (DHCA) and trihydroxycholestanoic acid (THCA) can accumulate [2, 4]. These intermediates are thought to contribute to liver disease and are possibly neurotoxic [2, 3, 4, 10, 11, 12, 13, 14, 15, 16].

CA treatment is currently authorized in the European Union (EU) as an orphan drug for the indications 3β‐hydroxy‐Δ^5^‐C_27_‐steroid oxidoreductase (3β‐HSD) and Δ^4^‐3‐oxosteroid‐5β‐reductase (AKR1D1) deficiency [17, 18]. Until 2020, CA was also authorized in the EU for the indications sterol 27‐hydroxylase deficiency (causing cerebrotendinous xanthomatosis, CTX), α‐methylacyl‐CoA racemase (AMACR) deficiency, and cholesterol 7α‐hydroxylase (CYP7A1) deficiency. However, this authorization was withdrawn at the request of the second authorization holder [19, 20]. Furthermore, CA treatment has not been available for Dutch BASD patients by either EU registration holder. To our knowledge, CA is currently not available in other EU countries.

The limited availability of clinical trial data has also contributed to the reluctance of healthcare professionals in the Netherlands to consider CA treatment as part of standard care of BASD. To address this and generate clinical evidence, we developed and produced CA capsules for use in this clinical study [21]. The aim of this study is to assess the safety, biochemical, and clinical effectiveness (based on surrogate outcome measures) of CA treatment in BASD patients, including six individuals (three adults and three children) with AMACR deficiency and one individual with 3β‐HSD deficiency [22]. This report presents the results of 3.5 years follow‐up.

## 2 Methods

### 2.1 Study Design

Patients with a 3β‐HSD, AKR1D1, AMACR, and CYP7A1 deficiency and at least one hallmark (steatorrhea, elevated transaminases, intellectual disability, and/or other neurological symptoms) were eligible for inclusion (in correspondence with the EU registration of CA). Additionally, patients with ZSD were eligible for inclusion (consistent with the FDA approval of CA). CTX patients were excluded from this study, since CDCA is the first‐choice treatment for CTX in the Netherlands [23]. The study was approved by the Institutional Review Board of the Amsterdam University Medical Centers (Amsterdam UMC).

Individual written informed consent was obtained from the patients and/or legal guardians. Patients were seen every 3 months during the first treatment year and every 6 months thereafter. Additional visits were scheduled 1 month after dose adjustments or as needed based on clinical judgment. Additional follow‐up via telephone or email was arranged when indicated. Blood and urine samples were collected for biochemical analyses, and a standard physical and neurological examination was performed during each visit. An ultrasound (US) of the liver was performed at the start of CA treatment and was repeated when indicated. A fibroscan or US elastography was performed at the start of CA treatment and was repeated yearly. For AMACR deficiency patients, brain MRI and an ophthalmologic examination were performed at the start of treatment and were repeated after 2 years, after which they were repeated when indicated.

The primary objective was to investigate the long‐term safety of personalized CA treatment of patients with the specified BASDs on clinical parameters (occurrence of adverse events [AEs] and side effects, including negative effects on the liver) and biochemical parameters (defined by change in plasma aspartate aminotransferase [AST], alanine aminotransferase [ALT], conjugated bilirubin, and the degree of suppression of C_27_‐bile acid intermediates DHCA and THCA in plasma and urine).

The secondary objectives were (1) to investigate the long‐term effect of CA treatment, including biochemically relevant parameters (i.e., liver tests and protein synthesis, liver elasticity, fatty acids, fat‐soluble vitamin absorption, and growth), neurological and clinical assessments, and (2) to determine the feasibility of personalized treatment based upon these clinical‐ and biochemical parameters.

### 2.2 CA Treatment

CA capsules were developed and manufactured according to the guidelines of Good Manufacturing Practice (GMP) by the pharmacy of Amsterdam UMC [21, 24]. CA was administered orally in initial doses of 15 mg/kg/day once daily or divided into two or three doses per day, depending on patient preference, daily routine, and tolerability. In case of side effects, the dose was divided over more intakes. If adherence was challenging, the intake schedule was simplified.

Personalized daily dosing was determined based on the degree of suppression of the concentrations of the toxic C_27_‐bile acid intermediates THCA and DHCA, the occurrence of side effects, and clinical evaluation. In case a patient was overweight, the daily dose was calculated using an ideal body weight (body mass index [BMI] between 19 and 25). The best tolerable CA dose was chosen that effectively reduced the THCA and DHCA plasma levels to as close to zero as possible. In case necessary due to side effects, the dose was reduced in steps of 33% to 10 or 5 mg/kg/day. Temporary treatment cessation was allowed, followed by treatment rechallenge after the side effects had subsided. During rechallenge, the treatment dose was increased gradually in small steps with a minimum of 1 mg/kg every couple of days.

### 2.3 Biochemical Analysis, Liver Stiffness Measurements, and Physical Examination

Plasma and urinary bile acids were measured by the Laboratory Genetic Metabolic Diseases in the Amsterdam UMC, as described by Bootsma et al. [25]. The lower detection limit of bile acid intermediates in this assay is 0.01 μmol/L. A target value of as close to zero as possible was set for plasma C_27_‐bile acid intermediate levels. Urinary bile acids were measured qualitatively only, and comprised primary bile acids (conjugates), bile alcohols, and C_27_‐bile acid intermediates based on the method of Leníček et al. [26]. Standard diagnostic assays were used to measure levels of conjugated bilirubin, plasma transaminases, fat‐soluble vitamins, total cholesterol, albumin, and coagulation factors (i.e., prothrombin time [PT], activated partial thromboplastin time [aPTT]).

Liver stiffness analyses were performed at baseline and were repeated yearly by trained physicians using US elastography or transient elastography (FibroScan), according to the standard manufacturer instructions (Echosens, Paris, France). Severe liver fibrosis or cirrhosis was defined as a FibroScan value ≥ 15.5 kPa [27].

Weight was measured with a calibrated balance, and age‐ and gender‐specific standard deviation (SD) scores were calculated using current Dutch reference values [28].

For AMACR deficiency patients, brain MRI analysis was performed by trained radiologists per local protocol. Additionally, ophthalmological examination was performed according to local protocol (optical coherence tomography [OCT] and fundus autofluorescence [FAF]) by an experienced ophthalmologist.

### 2.4 Monitoring and Safety Assessment

Side effects and (serious) AEs ((S)AEs) were assessed during each follow‐up visit based on clinical judgment. Side effects were defined as any new or worsening symptoms for which a causal relationship with CA treatment was suspected or considered plausible by the treating physician. In the assessment of gastrointestinal side effects, new or clearly aggravated symptoms—particularly diarrhea—were evaluated relative to the patient's baseline status. Occasional use of over‐the‐counter medications such as laxatives or antacids at baseline was not associated with formal GI diagnoses and was not considered indicative of underlying GI disease. Symptoms without such a suspected relationship were recorded as (S)AEs.

A symptom or complaint was considered a new side effect or (S)AE only if a similar symptom had previously resolved and reappeared during treatment. Attribution to CA treatment was based on temporal relationship, absence of an alternative explanation, and response to dose adjustment if applicable.

Although diarrhea and pruritus can be clinical manifestations of the underlying BASD [29], they have also been reported as known side effects of CA therapy [18, 30, 31]. In our study, if these symptoms were absent prior to treatment initiation and occurred after starting CA, they were classified as possible side effects.

### 2.5 Statistical Analysis

Data analyses were performed with GraphPad Prism software 10.2.0. Due to the rarity of the studied metabolic disorders, the patient group was not large enough to allow robust statistical analysis.

## 3 Results

### 3.1 Patient Characteristics

Eight patients were screened for participation in the study. Seven patients were ultimately included in the study. Six patients had an AMACR deficiency (#1–5 and #7), three of whom were children (#1–3). One patient had a 3β‐HSD deficiency (#8), and one patient with ZSD (#6) who was excluded due to screen failure (baseline plasma DHCA and THCA levels were below 1.0 μmol/L, which met the exclusion criterion).

All AMACR patients followed a phytanic acid‐restricted diet as part of standard care to minimize accumulation of pristanic and phytanic acid. This dietary guidance was provided by a dietitian and ensured adequate caloric intake. None of the patients required additional caloric supplementation. Patients #2 and #3 had previously received CDCA at doses of 250 mg once and twice daily, respectively. This treatment was discontinued approximately 5 years prior to the current trial due to concerns about CDCA's potential hepatotoxicity in patients with preexisting liver dysfunction.

Patient #8 had been previously treated with CDCA and then CA, but was ultimately treated with glycodeoxycholic acid (gDCA), 400 mg per week, which maintained a stable biochemical and clinical profile for 8 years prior to this study. CA treatment was initiated without a washout period between treatments [32].

Two adult AMACR patients (#5 and #7) discontinued CA treatment due to gastrointestinal intolerance. The remaining five patients tolerated the treatment well with dose adjustments.

Most of the patients used fat‐soluble vitamin supplementation during CA treatment before participating in this study (Table 1). The older adult patients (#4, #5, and #7) also used other co‐medications such as antihypertensives, antiplatelet medication, bisphosphonates, nonsteroidal anti‐inflammatory drugs (NSAIDs), laxatives, and/or antacids for other indications than AMACR deficiency (Table 1). There are no known interactions between these medications and CA.

**TABLE 1:** Clinical and biochemical characteristics of participants treated with CA.

Patient #	Sex	Deficiency	Age at diagnosis (years)	Clinical findings at diagnosis	Age at start of CA treatment (years)	Weight at start of CA treatment (kg)	Hallmarks at study enrollment	Treatment duration (months)	CA dose (mg/kg)		Daily dosing frequency	Vitamin supplementation
									Start	Final		
1	M	AMACR	6	Listless and tired, elevated liver chemistries, elevated pristanic acid	10	44	– Elevated AST/ALT	42	15	10	2	A, D, E
2^a^	F	AMACR	3	No obvious clinical signs	8	21.5	– Elevated AST/ALT – Developmental delay – Neurological symptoms (nystagmus)	42	15	20	2	A, D, E
3^a^	M	AMACR	5	No obvious clinical signs	10	38.5	– Elevated AST/ALT – Neurological symptoms (nystagmus)	42	15	20	2	A, D, E
4	F	AMACR	62	Elevated liver chemistries, indications of retinopathy, cerebellar syndrome and polyneuropathy	63	80	– Neurological symptoms (cerebellar syndrome, polyneuropathy, retinitis pigmentosa, cognitive impairment)	42	15	15	3	D
5	F	AMACR	41	Difficulty walking, poor vision and night blindness, reduced ability to concentrate, tingling in hands and feet, mild dysarthria, mild ataxia, stationary abnormalities seen in the pons	43	67.7	– Neurological symptoms (cognitive impairment, reduced vision, wheelchair dependent, (mild) dysarthria, swallowing difficulties, pain complaints)	16	15	Dropped out	3	D
7	F	AMACR	58	Retinitis pigmentosa with encephalopathy, head tremor, paroxysmal hemicrania, familial gallstones, progressive headache	62	57	– Neurological symptoms (cognitive impairment)	10	15	Dropped out	3	—
8^b^	F	3β‐HSD	11	Failure to thrive, chronic pancreatitis, ataxia, steatorrhea	29	40.8	– Elevated AST/ALT – Developmental delay – Neurological symptoms (mild cerebellar ataxia, mild cognitive impairment)	12	15	20	1	D, K

Abbreviations: 3β‐HSD, 3β‐hydroxy‐Δ^5^‐C_27_‐steroid oxidoreductase; ALT, alanine aminotransferase; AMACR, α‐methylacyl‐CoA racemase; AST, aspartate aminotransferase; CA, cholic acid; F, female; M, male.

^a^ Siblings.

^b^ Previously treated with gDCA, no washout period.

Table 1 summarizes key patient demographics, clinical presentation (at diagnosis and baseline), study treatment characteristics, and use of vitamin supplementation.

### 3.2 Bile Acid Plasma‐ and Urine Levels

**FIGURE 1:**
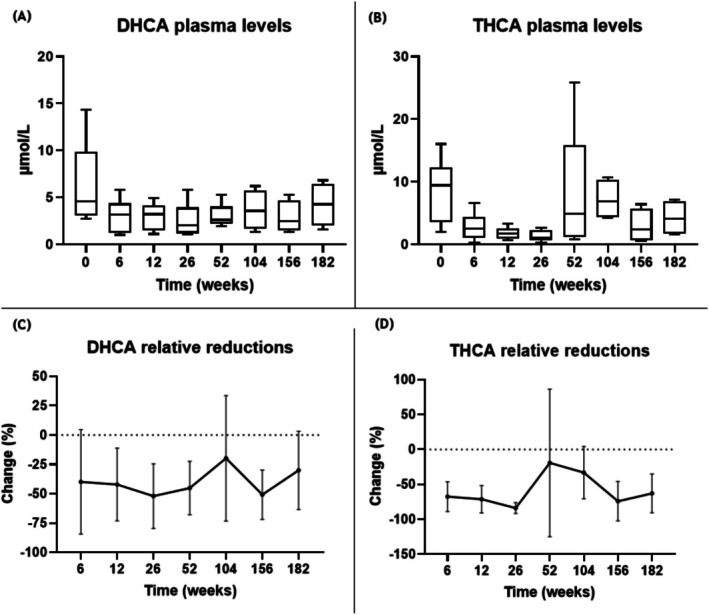
*Panels A and B*: Box plot (min to max) of DHCA (panel A) and THCA (panel B) plasma levels before (T0) and during CA treatment of AMACR patients (*n* = 6). *Panels C and D*: Line plot showing the percentage change in DHCA (panel C) and THCA (panel D) plasma levels relative to baseline during CA treatment of AMACR patients (*n* = 6). Data points represent mean values with error bars indicating the standard deviation. Negative values reflect reductions compared to baseline.

All AMACR deficiency patients had elevated DHCA and THCA plasma levels at the start of CA treatment (median: 3.6 and 9.3 μmol/L, respectively [reference range 0–0.02 μmol/L and 0–0.08 μmol/L, respectively]). CA treatment resulted in a marked reduction of these intermediates during the first year. However, inconsistent reductions in both intermediates were seen as plasma levels fluctuated during the second and third years of treatment (Figure 1).

Although fluctuations in DHCA and THCA plasma levels were recorded during CA treatment, overall plasma levels remained reduced compared to baseline for all patients (Table S1). Only one AMACR patient (#5) reached values < 1.0 μmol/L for THCA, which was maintained until this patient dropped out of the study due to persistent diarrhea (also see Section 3.4.1). The three AMACR children also reached values < 1.0 μmol/L for THCA at different time points; however, this was not maintained consistently throughout the 3.5 years of treatment.

In case of continued increased levels of DHCA and/or THCA, the dose of CA was increased to a maximum of 20 mg/kg. Adherence issues were addressed when elevated DHCA/THCA levels and relatively low CA plasma levels (reference range 0.1–4.7 μmol/L) were measured, and if preferred, the intake schedule was adjusted to increase treatment adherence. For example, patient #2 showed a marked increase in THCA levels at week 52, prompting dose adjustments and discussions around adherence to improve treatment outcomes.

At baseline, all AMACR patients had significantly elevated C_29_‐dicarboxylic acid (≥ 2 × ULN, reference range 0–0.001 μmol/L). C_29_ levels normalized in patients #2–#5 after 6 weeks of CA treatment and more than halved in patient #1. These levels remained decreased after 3.5 years in patients #1–#4 (Table S1).

The 3β‐HSD patient (#8), who was initially treated with gDCA and had low levels of typical ∆5‐bile acid intermediates [32], had increasing levels after switch to CA treatment, but was clinically stable throughout the study.

### 3.3 Liver Function

**FIGURE 2:**
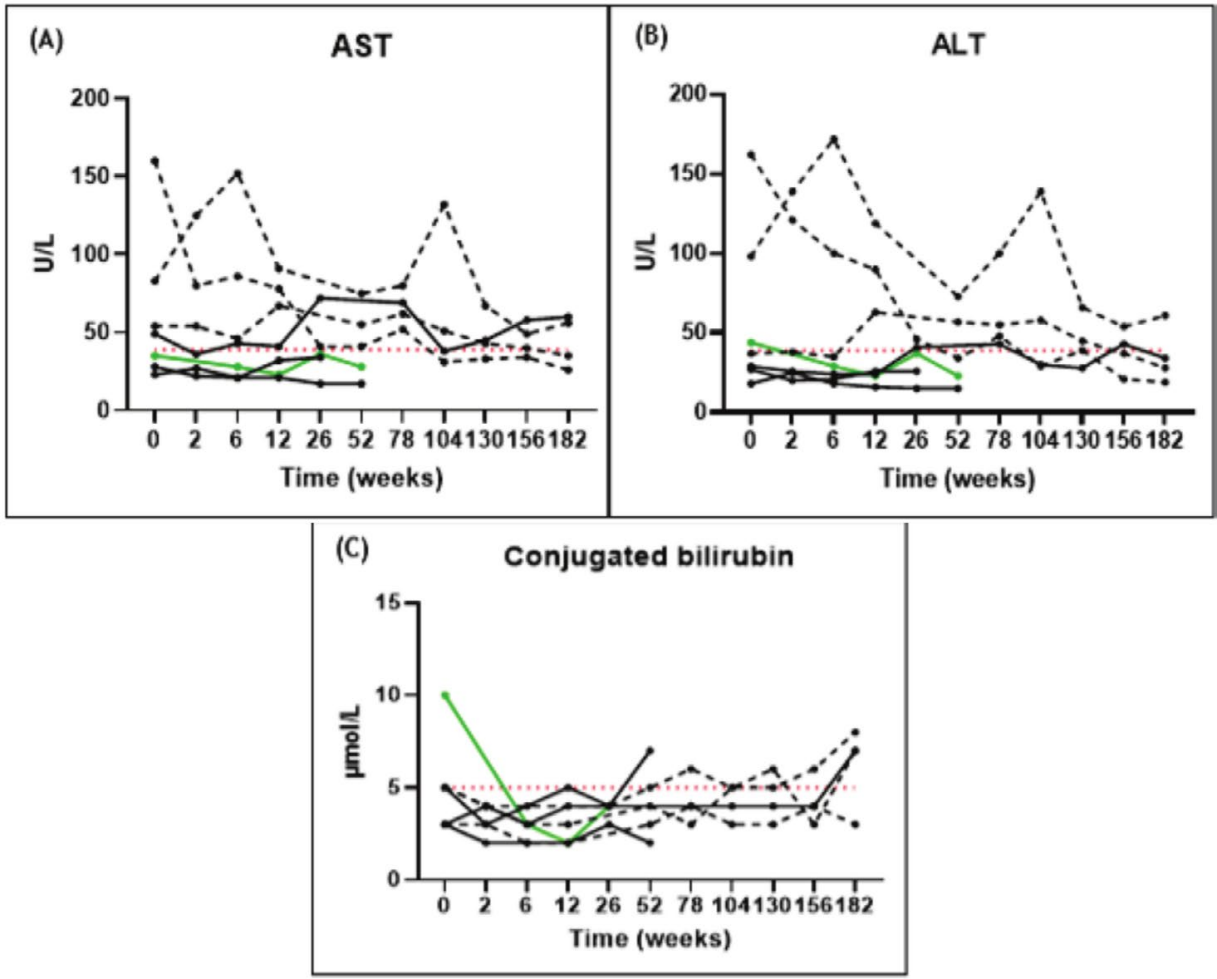
Graphs showing the individual courses of AST (panel A), ALT (panel B), and conjugated bilirubin (panel C) before (T0) and during CA treatment (*n* = 7). The patients with AMACR deficiency are depicted in black, the children are shown as black dotted lines. The 3β‐HSD patient is depicted in green. The upper control reference value is indicated by the red dotted line; AST (39 U/L), ALT (39 U/L), and conjugated bilirubin (5 μmol/L).

AST and ALT levels are presented in Figure 2A,B. These were elevated (≥ 2 × ULN) at baseline in two pediatric AMACR patients (#1 and #3) and had normalized after 3.5 years of CA treatment in patient #1, while they remained elevated in patient #3 (Table S2).

Baseline plasma level of conjugated bilirubin was elevated (≥ 2 × ULN) in the 3β‐HSD patient (#8), but normalized quickly after CA treatment was started (Table S2).

No clinically relevant changes were observed in liver parameters (total bilirubin, alkaline phosphatase [ALP], ɣ‐glutamyl transferase [GGT], α‐fetoprotein [AFP], coagulation factors FV or FVII, PT, aPTT, and total cholesterol). Plasma values for these parameters remained within the reference range throughout the study (Tables S2 and S3).

One patient (#4) developed hepatocellular carcinoma (HCC) after 3.5 years of CA treatment.

### 3.4 Side Effects and (S)AEs

#### 3.4.1 Side Effects

**FIGURE 3:**
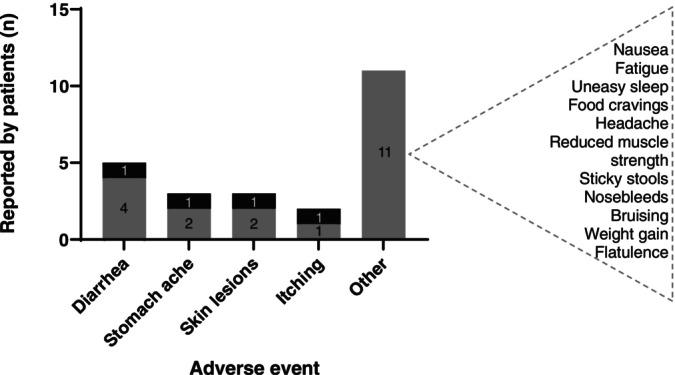
Reported side effects during CA treatment by patients (*n*); children (black) and adults (gray).

Diarrhea and abdominal pain were the most common side effects, reported by five patients (Figure 3). Some patients reported these side effects multiple times, as it could reoccur at different moments during treatment. Skin lesions were reported in three patients (i.e., small skin rash or bumps combined with dry skin and itching, managed effectively with topical treatments) probably unrelated to CA treatment. None of the patients who reported diarrhea or pruritus experienced these symptoms prior to the initiation of CA treatment.

Children tolerated CA better than adults, who reported more frequent and severe side effects. Temporary cessation of CA treatment resolved the complaints. To prevent recurrence, dose reductions were necessary for two adult and one pediatric patient. Treatment rechallenge with a lower CA dose often resulted in a (temporary) absence of these side effects. Despite dose reductions and careful treatment rechallenge, the two adult AMACR patients continued to experience mild to moderate side effects (i.e., persistent diarrhea and abdominal pains) at lower doses (7 mg/kg/day).

**FIGURE 4:**
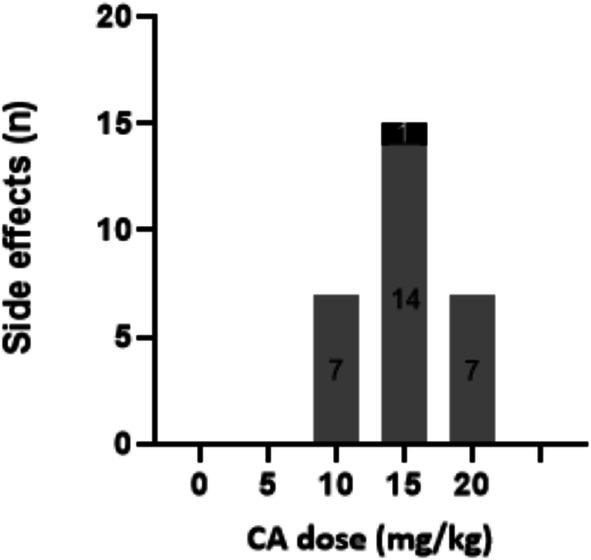
Number of reported side effects reported per CA dose category by children (black) versus adults (gray).

More side effects were reported for the adults and generally there was a less clear correlation between treatment dose and the occurrence of the side effects (Figure 4).

#### 3.4.2 (S)AEs

Eleven AEs were reported, all in adult AMACR patients. These included kidney stones, cold and flu symptoms, an arm fracture, neck pain, tingling sensations in hands and feet, hematuria and bladder spasms, and nonmalignant intestinal polyps (all *n* = 1). Most AEs were categorized as mild to moderate and were assessed as unrelated to CA treatment, and resolved without long‐term consequences.

Three SAEs occurred: one hospitalization for a bone fracture after a fall due to hypotension following significant weight loss (> 10%), one involving bladder carcinoma, treated with local chemotherapy, and another who developed a HCC was treated with ablation and was awaiting the possibility of liver transplantation. All SAEs were assessed as unrelated to CA treatment, but potentially due to the underlying disorder [33].

### 3.5 Secondary Outcome Measures

#### 3.5.1 Fatty Acids

Plasma levels of pristanic acid and phytanic acid were measured during CA treatment. At baseline, all AMACR patients had significantly elevated pristanic acid levels (≥ 2 × ULN, reference range 0–1.6 μmol/L). During CA therapy, these levels fluctuated and remained elevated, although a general decrease was seen (Table S4). Phytanic acid levels remained within the normal range (0.49–9.88 μmol/L) at all measured time points. All AMACR patients followed a phytanic acid‐restricted diet since before the study, which likely contributed to the stable phytanic acid levels.

#### 3.5.2 Fat‐Soluble Vitamin Absorption

No marked changes were observed in the plasma levels of fat‐soluble vitamins A, D, or E (Table S4). All patients required vitamin supplementation: adults needed vitamin D supplementation, and the children required vitamin A, D, and E supplementation (Table 1).

#### 3.5.3 Liver Stiffness

Liver stiffness measurements remained stable in most patients throughout the course of CA treatment. All values were below 7.1 kPa at 36 months, indicating no progression to liver fibrosis (F1 or lower). Patient #3 had a temporary increase to 7.1 kPa at 12 months but returned to 5.8 kPa by 36 months, while patient #4 showed a decrease from 9.0 to 7.2 kPa over time, suggesting improvement in liver stiffness. Patients #5 and #7 dropped out early, and patient #8 had incomplete data, but initial measurements were within normal limits (Table 2).

**TABLE 2:** Liver stiffness measurements from patients treated with CA (Table 2).

CA treatment	Liver stiffness (kPa)			
	0 months	12 months	24 months	36 months
Patient #				
1	4.29	4.38	4.1	4.9
2^a^	N/A	6.48	4.1	3.1
3^a^	N/A	5.93	7.1	5.8
4	N/A	9.0	6.2	7.2
5	3.9	3.4	Dropped out	
7	4.6	Dropped out		
8^b^	4.9	…	…	…

Abbreviations: kPa, kilopascals; N/A, no value available, but no abnormalities seen. Interpretation, Fibroscore estimation based on liver elasticity measurement (< 7.1 kPa [F1]; ≥ 7.1 to < 9.5 kPa [F2]; ≥ 9.5 to < 12.5 kPa [F3]; ≥ 12.5 kPa [F4])[34].

^a^ Siblings.

^b^ Previously treated with gDCA, no washout period. …: time point has not been reached yet.

#### 3.5.4 Growth in AMACR Children

**FIGURE 5:**
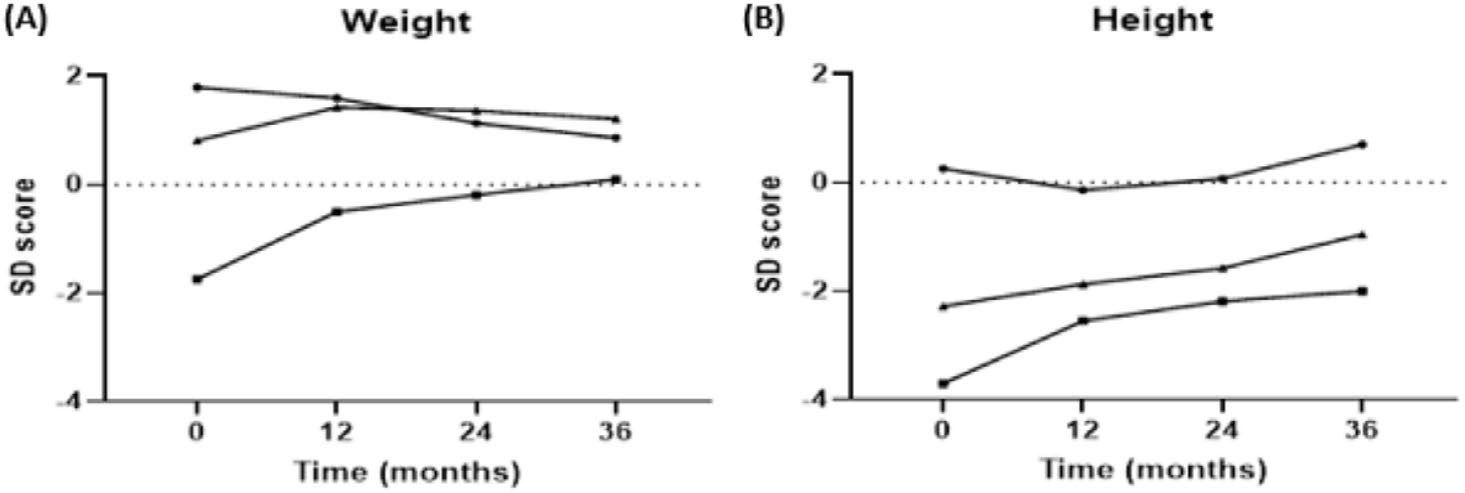
Change standard deviation (SD) scores for weight (panel A) and height (panel B) of children with AMACR deficiency during CA treatment. SD scores were calculated using current Dutch reference values. Symbols: patient #1 (●), patient #2 (■), and patient #3 (▲).

In the three children with AMACR deficiency, growth parameters improved during CA treatment, as shown by their weight and height SD scores (Figure 5). Patient #2, who had a significant growth retardation at baseline (SD −1.74 for weight and SD −3.70 for height), showed marked improvement, reaching near‐normal weight (SD +0.10) after 36 months. Patients #2 and #3 (siblings), both smaller than average at the start of treatment, showed improved height SD scores after 36 months (from SD −3.70 to SD −2.00 and from SD −2.28 to SD −0.96, respectively). The changes in weight and height for patient #1 were minimal and not clinically relevant.

#### 3.5.5 Neurological Assessment in AMACR Patients: Brain and Retinal Abnormalities

None of the three children showed brain abnormalities either before or after 2 years of CA treatment. All adults had brain abnormalities consistent with AMACR deficiency at baseline and remained stable during treatment.

Retinal abnormalities were observed in all patients except for patient #1 at baseline. Patients #2 and #4 remained stable during CA treatment. Patient #3 developed early cataracts in one eye after 2 years of treatment.

In all patients, the neurological symptoms found at baseline (Table 1) remained stable during treatment.

## 4 Discussion

In this study, we evaluated the long‐term safety, biochemical, and change in surrogate outcomes of personalized CA treatment in patients with BASDs, specifically AMACR and one patient with a 3β‐HSD deficiency. Dose titration was a critical aspect of the treatment protocol. The initial dose of 15 mg/kg/day was adjusted based on the suppression of DHCA and THCA levels and observed side effects. In children, dose titration led to higher doses, better control of bile acid intermediates, and fewer side effects. However, in adults, side effects limited further titration, preventing the achievement of optimal therapeutic levels. This reinforces the need for individualized dosing strategies and careful monitoring, especially in adults due to a seemingly narrower therapeutic window. The most commonly reported side effect was diarrhea, sometimes with severe abdominal pain, which often resolved upon dose adjustment. Diarrhea and pruritus are common side effects reported for CA [17, 18, 19, 30, 31].

CA treatment led to marked reductions of C_27_‐bile acid intermediates (THCA and DHCA), with percentage reductions often exceeding 50%, particularly in children. However, absolute values decreased only marginally compared to the upper limit of normal (ULN; 0.08 and 0.02 μmol/L, respectively), especially in adults. This raises questions about the clinical relevance of these biochemical changes. The limited ability to achieve normalization, combined with side‐effect‐related dose limitations in adults, highlights the need for a better understanding of the relationship between biochemical response and clinical outcomes. In view of the above and given the small sample size, particularly for the 3β‐HSD patient, these observations should be considered exploratory.

Achieving target levels for THCA and DHCA (as close to zero as possible) was not achieved, although levels ≤ 1.0 μmol/L were reached inconsistently in all patients but one (#4), particularly in the second and third years of treatment. The extent of suppression was largely dependent on baseline levels, with greater reductions observed in patients with higher THCA and DHCA baseline values. This supports considering relative reductions rather than absolute target values when evaluating treatment effectiveness. Moreover, CA impacted THCA levels more than DHCA.

In the case of the 3β‐HSD patient (#8), elevated DHCA and THCA levels were not observed nor expected. 3β‐HSD deficiency affects an earlier step in bile acid synthesis, leading to the accumulation of ∆5‐bile acid intermediates rather than the downstream products DHCA and THCA, typically seen elevated in peroxisomal disorders like AMACR deficiency [4]. After 1 year of treatment—and compared to previous gDCA treatment—a qualitative increase in these intermediates was observed, suggesting that CA might not be sufficiently effective in inhibiting the formation of these metabolites. This observation is consistent with the fact that gDCA is a more potent FXR agonist compared to CA. It appears CA does not suppress bile acid synthesis as effectively as gDCA did in this patient, which would explain the observed increase in ∆5‐bile acid intermediates during CA treatment. However, since this conclusion is based on a single case, no definitive statement can be made about CA's general effectiveness in 3β‐HSD deficiency. Although previous studies have reported positive biochemical responses to CA in 3β‐HSD deficiency, interpretation is limited due to lack of pre‐ and post‐treatment comparison or generalization across BASD subtypes [29, 35, 36, 37, 38]. Further studies in larger cohorts are needed to clarify the role of CA in managing 3β‐HSD deficiency.

In regards to growth in the children with AMACR deficiency, it is difficult to draw a definite conclusion regarding the effects of CA. Aside from the small group, two of the children are siblings who also have albinism and are of non‐Dutch ethnicity, making the SD scores (calculated according to Dutch reference values) challenging to interpret.

Treatment adherence issues arose mainly in children, with midday doses often missed on school days. Dividing the daily dose over two intake moments improved adherence. Increased DHCA and THCA plasma levels signaled possible adherence issues, prompting family consultations.

No clinically relevant changes were observed in liver enzyme levels, liver elasticity, or fat‐soluble vitamin absorption. All patients continued to require fat‐soluble vitamin supplementation throughout the study, and levels remained low. Elevated transaminases (AST/ALT) were observed at the start of CA treatment in some patients but normalized and remained stable with dose optimization. However, it is possible that the observed normalization of liver enzymes is attributable to the natural course of childhood development, taking into account the case report by Gündüz et al. [39], who also described spontaneous normalization of transaminase levels with age in a child with AMACR deficiency. No liver toxicity was observed, supporting the safety of CA treatment in this cohort. Patient #4, who developed HCC after 3.5 years of CA treatment, showed a limited biochemical response to CA. We believe that it is unlikely that the HCC was a consequence of potential liver toxicity of CA treatment. The development of HCC in this patient is more likely attributable to the underlying AMACR deficiency and its associated (prolonged) accumulation of toxic C_27_‐bile acid intermediates. Notably, this patient initiated CA therapy at age 63, suggesting that treatment, if effective, may have been started too late to prevent long‐term hepatic sequelae such as HCC. This interpretation is supported by the case of the patient's brother, who succumbed to HCC and was later evaluated for AMACR deficiency but was never treated with CA. Moreover, HCC in AMACR deficiency has been previously reported, with evidence suggesting a risk of liver fibrosis, cirrhosis, and eventual HCC in affected patients [40, 41]. The HCC diagnosis of patient #4 underscores that CA treatment does not prevent all liver complications [33].

Assessing the long‐term clinical effectiveness of CA treatment in AMACR and 3β‐HSD deficiencies is challenging due to slow disease progression and late onset of neurological symptoms and gradual deterioration, often emerging in adulthood [2, 4, 39, 41, 42, 43, 44, 45]. Although early biochemical improvements were observed in the children with AMACR, it remains uncertain whether these translate into long‐term clinical benefit. Long‐term follow‐up is therefore essential to assess CA's potential effects on liver and neurological outcomes.

Similarly, in the 3β‐HSD patient (18 years), slow disease progression limits conclusions on long‐term effectiveness [2, 4]. While early biochemical responses were observed, their clinical significance remains to be determined.

This study is limited by its small sample size, lack of a control group, patient heterogeneity, and relatively short follow‐up period (3.5 years), which is insufficient to detect long‐term clinical outcomes in these slowly progressive disorders. Moreover, factors such as dietary interventions (e.g., phytanic acid restriction in AMACR) were not standardized and may have influenced biochemical or clinical parameters.

To better assess CA treatment effectiveness, standardized long‐term follow‐up monitoring strategies, tailored to BASD subtype, are needed. These should include predefined clinical outcome measures for hepatic (e.g., liver transaminases and liver stiffness) and neurological status (e.g., standardized MRI protocols and cognitive assessments), as well as biomarkers such as C_27_‐bile acid intermediates. Data from case reports and small cohorts could be used more effectively through a centralized, independent disease registry, enabling pooled analyses and better insight into disease progression and treatment outcomes. Until such tools are in place, interpretation of biochemical and clinical responses should remain cautious.

## 5 Conclusion

This study demonstrates that personalized CA treatment is generally safe and moderately tolerated in patients with BASDs such as AMACR‐ and 3β‐HSD deficiencies. In children, CA treatment was better tolerated and resulted in biochemical improvements, particularly the partial suppression of C_27_‐bile acid intermediates indicating that bile acid synthesis is still not sufficiently suppressed. In adults, the balance between biochemical efficacy and side effects was particularly unfavorable, as side effects restricted dose escalation needed to achieve target biochemical outcomes. Personalized dosing adjustments and careful monitoring are essential to optimize treatment outcomes, where higher dosages per kilogram body weight are tolerated in children and lower dosages for adults must be taken into account due to side effects. The combination of partial biochemical response and its difficult‐to‐interpret clinical relevance makes it difficult to make firm statements about the effect of CA treatment on the clinical course of AMACR deficiency. For the treatment of the patient with 3B‐HSD deficiency, CA treatment appears to be insufficiently effective to inhibit the formation of toxic metabolites.

## Author Contributions

Y.P., E.M.K., M.E., F.C.C.K., K.B., F.M.V., B.G.P.K., and C.E.M.H. contributed to the conception and design of the study. Y.P., F.M.V., and C.E.M.H. contributed to the analysis of the data. All authors contributed to the interpretation of the data. Y.P. drafted the article. All other authors revised the article critically for important intellectual content. C.E.M.H. (Guarantor) provided overall supervision of the project. All authors read and approved the final version of the manuscript and agree with submission. This work has not been published/submitted elsewhere.

## Ethics Statement

The trial was approved by the Institutional Review Board of the Amsterdam University Medical Centers (Amsterdam UMC).

## Consent

All procedures followed were in accordance with the ethical standards of the responsible committee on human experimentation (institutional and national) and with the Helsinki Declaration of 1975, as revised in 2000 (5). Informed consent was obtained from all patients for being included in the study.

## Conflicts of Interest

E.M.K. is a board member of the Dutch Hospital Pharmacist Association. She receives no personal fees, and the association has no involvement in the preparation of this manuscript or any interest in the product or patients described. M.E. has received research grants from ZonMw, Spur Therapeutics, and Minoryx to perform clinical studies outside the scope of this manuscript and has received consulting fees from Spolia. He received no personal fees. F.M.V. has served as a consultant for Scenic Biotech, is a board member of the European Metabolic Group (EMG), secretary of the SSIEM Education and Training Advisory Committee (ETAC), and is an inventor of a patent for a screening method of cerebrotendinous xanthomatosis (PCT/EP2018/055236). E.L.S. has received personal fees for expert assistance in a patent lawsuit by Pinsent Masons and is chair of the scientific advisory board for the Centre for Human Drug Research (CHDR), for which she receives no fees. She is chair of the scientific advisory board Commissie Farmacotherapeutisch Kompas at the Zorginstituut and a member of the Committee on Vitamin K in Newborns at the Health Council of the Netherlands, for both of which she receives personal fees. She is also chair of the Dutch Society of Clinical Pharmacology and Biopharmacy (no fees received). C.E.M.H. has received grants from Sanofi, Idorsia, and Protalix for clinical studies in the field of lysosomal storage disorders, which are unrelated to the current manuscript. She received no personal fees. The remaining authors declare no conflicts of interest.

## Supporting information


**Table S1.** Biochemical analyses in plasma and urine of AMACR (#1–7) and 3β‐HSD (#8) patients treated with CA.

**Table S2.** Liver function tests of AMACR (#1–7) and 3β‐HSD (#8) patients treated with CA.

**Table S3.** Coagulation factors and cholesterol tests of AMACR (#1–7) and 3β‐HSD (#8) patients treated with CA.

**Table S4.** Plasma tests of long chain fatty acids, phytanic acid, pristanic acid, and fat‐soluble vitamins of AMACR (#1–7) and 3β‐HSD (#8) patients treated with CA.

## Data Availability

The data of this study has been included as electronic supplementary material.
