# Supplementary material for: Effectiveness and Safety of Personalized Cholic Acid Treatment in Patients With Bile Acid Synthesis Defects
Source: J Inherit Metab Dis. 2025 Jul 11;48(4):e70062. doi: 10.1002/jimd.70062 (PMC12247693; doi:10.1002/jimd.70062)
Supplement: Supplementary file 1 — Table S1. Biochemical analyses in plasma and urine of AMACR (#1–7) and 3β‐HSD (#8) patients treated with CA. [file JIMD-48-0-s001.docx]

Supplementary Table 1. Biochemical analyses in plasma and urine of AMACR (#1-7) and 3β-HSD (#8) patients treated with CA

| CA treatment | Week 0 | | | | | | Week 6 | | | | | | Week 12 | | | | | | Week 26 | | | | | |
| --- | --- | --- | --- | --- | --- | --- | --- | --- | --- | --- | --- | --- | --- | --- | --- | --- | --- | --- | --- | --- | --- | --- | --- | --- |
| Patient # | **CA** | **CDCA** | **DHCA** | **THCA** | **C29** | **Urine^¥^** | **CA** | **CDCA** | **DHCA** | **THCA** | **C29** | **Urine^¥^** | **CA** | **CDCA** | **DHCA** | **THCA** | **C29** | **Urine^¥^** | **CA** | **CDCA** | **DHCA** | **THCA** | **C29** | **Urine^¥^** |
| 1 | 1.0 | 3.0 | 2.7 | 9.3 | 0.7 | + | 5.1 | 3.4 | 3.8 | 6.6 | 0.2* | NA | 2.6 | 1.2 | 1.6 | **0.7*** | 0.2* | NA | 5.3 | 2.9 | 1.2* | 2.1* | nd* | - |
| 2^a^ | 0.6 | 0.7 | 3.2 | 9.6 | 0.3 | + | 7.9 | 0.8 | **1.0*** | 1.3* | nd* | + | 22.0 | 2.0 | 1.1* | 3.3* | nd* | + | 1.7 | 0.1 | 1.1* | **0.9*** | nd* | - |
| 3^a^ | 0.4 | 0.5 | 14.3 | 11.1 | 0.2 | + | 4.1 | 0.9 | 3.9* | 3.7* | nd* | - | 2.4 | 0.6 | 2.6* | 2.2* | NA | - | 1.5 | 0.1 | 2.3* | **0.8*** | nd* | - |
| 4 | 0.4 | 1.3 | 8.4 | 16.0 | 0.6 | + | 16.3 | 1.3 | 5.8 | 3.5* | nd* | + | 6.7 | 0.4 | 4.9 | 1.4* | nd* | + | 13.1 | 1.3 | 5.8 | 2.6* | nd* | - |
| 5 | 0.2 | 0.6 | 5.5 | 2.0 | 0.5 | + | 5.4 | 0.6 | 1.3* | **0.3*** | nd* | + | 9.5 | 1.3 | 3.8 | **1.0*** | nd* | - | 5.2 | 0.5 | 1.8* | **0.3*** | NA | - |
| 7 | 0.1 | 0.5 | 3.6 | 4.0 | NA | - | 1.3 | 0.3 | 2.5 | 1.6* | 0.1 | - | 0.1 | 0.3 | 3.9 | 2.1 | 0.3 | + | 5.4 | 0.4 | 3.3 | 1.1* | nd | + |
| 8 | 0.4 | 0.2 | **0.0** | **0.0** | nd | + | 28.1 | 0.2 | **0.0** | **0.0** | NA | + | 1.6 | 0.2 | **0.0** | **0.0** | nd | + | 6.9 | 0.3 | **0.0** | **0.0** | NA |  |
| *Median* | *0.4* | *0.6* | *3.6* | *9.3* | *0.4* |  | *5.4* | *0.8* | *2.5* | *1.6* | *0.0* |  | *2.6* | *0.6* | *2.6* | *1.4* | *0.0* |  | *5.3* | *0.4* | *1.8* | *0.9* | *0.0* |  |

Supplementary Table 1. *Continued*

| CA treatment | Week 52 | | | | | | Week 104 | | | | | | Week 156 | | | | | | Week 182 | | | | | |
| --- | --- | --- | --- | --- | --- | --- | --- | --- | --- | --- | --- | --- | --- | --- | --- | --- | --- | --- | --- | --- | --- | --- | --- | --- |
| Patient # | **CA** | **CDCA** | **DHCA** | **THCA** | **C29** | **Urine^¥^** | **CA** | **CDCA** | **DHCA** | **THCA** | **C29** | **Urine^¥^** | **CA** | **CDCA** | **DHCA** | **THCA** | **C29** | **Urine^¥^** | **CA** | **CDCA** | **DHCA** | **THCA** | **C29** | **Urine^¥^** |
| 1 | 2.0 | 1.8 | 1.9 | 1.5* | nd* | - | 3.4 | 4.5 | 2.8 | 4.3* | 0.1* | + | 0.9 | 0.4 | 1.3* | **0.5*** | 0.1* | + | 2.9 | 3.2 | 3.2 | 7.1 | 0.1* | + |
| 2^a^ | 9.3 | 2.5 | 2.4 | 25.8 | 0.1* | - | 1.9 | 1.0 | 4.3 | 10.7 | 0.1* | - | 18.4 | 1.1 | 2.1 | 6.4 | nd* | + | 3.1 | 0.3 | 1.6 | 1.6 | 0.1* | NA |
| 3^a^ | 2.4 | 0.4 | 2.7* | 4.9* | nd* | - | 3.4 | 1.2 | 1.3* | 9.2 | 0.1* | - | 7.9 | 0.1 | 2.8* | 1.1* | nd* | + | 0.9 | 0.3 | 6.8 | 1.9 | nd* | NA |
| 4 | 10.6 | 1.0 | 5.3 | 5.8* | NA | - | 7.5 | 1.0 | 6.2 | 4.5* | 0.1 | - | 5.6 | 0.4 | 5.3 | 3.6* | NA | + | 11.1 | 1.1 | 5.3 | 6.2 | 0.1 | NA |
| 5 | 9.0 | 0.6 | 2.6* | **0.8*** | nd* | - | *Dropped out* | | | | | | | | | | | | | | | | | |
| 7 | *Dropped out* | | | | | | | | | | | | | | | | | | | | | | | |
| 8 | 1.0 | 0.4 | 0.0 | 0.0 | .. | + | .. | .. | .. | .. | .. | .. | .. | .. | .. | .. | .. | .. | .. | .. | .. | .. | .. | .. |
| *Median* | *3.4* | *1.0* | *2.6* | *4.9* | *0.0* |  | *3.4* | *1.1* | *3.6* | *6.9* | *0.1* |  | *6.8* | *0.4* | *2.5* | *2.4* | *0.0* |  | *2.9* | *1.1* | *3.2* | *1.9* | *0.1* |  |

^a^ Siblings. *Abbreviations*: CA: cholic acid, CDCA: chenodeoxycholic acid, DHCA: dihydroycholestanoic acid, THCA: trihydroycholestanoic acid, C29: C29-galdicarboxylic acid, nd: not detected, NA: data not available. **^¥^**Urinary bile acids comprises C_27_-bile acid intermediates giving an abnormal urinary bile acid profile (+), or urinary C_27_-bile acid intermediates are not present, giving a normal urinary bile acid profile (-). Plasma bile acids are presented as the sum of unconjugated and conjugated bile acids in µmol/L, urinary bile acids are only measured qualitatively. *Reference range*: CA: 0.1-4.7 µmol/L, CDCA: 0.7-10 µmol/L, DHCA: 0-0.02 µmol/L, THCA: 0-0.08 µmol/L., C29: 0-0.001 µmol/L. Note: Significance of bold: DHCA/THCA value is ≤ 1.0 µmol/L. Significance of underlined: value is ≥ 2 x baseline value, *: value is ≤ 0.5 x baseline value. Time point has not yet been reached (..).
