# Supplementary material for: Effectiveness and Safety of Personalized Cholic Acid Treatment in Patients With Bile Acid Synthesis Defects
Source: J Inherit Metab Dis. 2025 Jul 11;48(4):e70062. doi: 10.1002/jimd.70062 (PMC12247693; doi:10.1002/jimd.70062)
Supplement: Supplementary file 2 — Table S2. Liver function tests of AMACR (#1–7) and 3β‐HSD (#8) patients treated with CA. [file JIMD-48-0-s004.docx]

Supplementary Table 2. Liver function tests of AMACR (#1-7) and 3β-HSD (#8) patients treated with CA

| CA  treatment | Week 0 | | | | | | | Week 2 | | | | | | | Week 6 | | | | | | |
| --- | --- | --- | --- | --- | --- | --- | --- | --- | --- | --- | --- | --- | --- | --- | --- | --- | --- | --- | --- | --- | --- |
| Patient # | **TB** | **CB** | **ALP** | **GGT** | **AST** | **ALT** | **AFP** | **TB** | **CB** | **ALP** | **GGT** | **AST** | **ALT** | **AFP** | **TB** | **CB** | **ALP** | **GGT** | **AST** | **ALT** | **AFP** |
| 1 | 12 | 5 | 249 | 15 | **160** | **162** | 2 | 12 | 4 | 260 | 14 | **80** | **121** | 2 | NA | NA | 222 | 15 | **86** | **100** | NA |
| 2^a^ | 5 | 3 | 160 | 8 | 54 | 37 | 1 | 6 | 3 | 228 | 11 | 54 | 38 | 1 | 4 | 2 | 218 | 11 | 46 | 35 | <1 |
| 3^a^ | 11 | 5 | 229 | 16 | **83** | **98** | 1 | 11 | 4 | 213 | 22 | **125** | **139** | 1 | 7 | 3 | 248 | 32 | **152** | **172** | 1 |
| 4 | 12 | 5 | 78 | 19 | 49 | 29 | 6 | 8 | 3 | 74 | 23 | 36 | 26 | 6 | 12 | 4 | 75 | 24 | 43 | 24 | 6 |
| 5 | 6 | 3 | 70 | 12 | 23 | 18 | 2 | 4 | 2 | 72 | 11 | 27 | 25 | 2 | 4 | 2 | 72 | 11 | 21 | 18 | 2 |
| 7 | 7 | 3 | 59 | 24 | 28 | 27 | 4 | 10 | 4 | 51 | 27 | 22 | 20 | 3 | 7 | 3 | 59 | 25 | 21 | 21 | 4 |
| 8 | 17 | **10** | 114 | 15 | 35 | 44 | 2 | NA | NA | NA | NA | NA | NA | NA | 4 | 3 | 99 | 47 | 28 | 29 | 2 |
| *Median* | *11.0* | *5.0* | *114.0* | *15.0* | *49.0* | *37.0* | *2.0* | *9.0* | *3.5* | *143.5* | *18.0* | *45.0* | *32.0* | *2.0* | *5.5* | *3.0* | *99.0* | *24.0* | *43.0* | *29.0* | *2.0* |

Supplementary Table 2. *Continued*

| CA  treatment | Week 12 | | | | | | | Week 26 | | | | | | | Week 52 | | | | | | |
| --- | --- | --- | --- | --- | --- | --- | --- | --- | --- | --- | --- | --- | --- | --- | --- | --- | --- | --- | --- | --- | --- |
| Patient # | **TB** | **CB** | **ALP** | **GGT** | **AST** | **ALT** | **AFP** | **TB** | **CB** | **ALP** | **GGT** | **AST** | **ALT** | **AFP** | **TB** | **CB** | **ALP** | **GGT** | **AST** | **ALT** | **AFP** |
| 1 | NA | NA | 242 | 15 | **78** | **90** | NA | 11 | 4 | 229 | 15 | 41 | 46 | 1 | 12 | 5 | 211 | 12 | 41 | 34 | 1 |
| 2^a^ | 5 | 2 | 232 | 13 | 67 | 63 | 1 | NA | NA | NA | NA | NA | NA | NA | 5 | 3 | 249 | 14 | 55 | 57 | 1 |
| 3^a^ | 8 | 3 | 238 | 28 | **91** | **119** | <1 | NA | NA | NA | NA | NA | NA | NA | 12 | 4 | 216 | 23 | 75 | **73** | 1 |
| 4 | 14 | 5 | 72 | 25 | 41 | 24 | 6 | 13 | 4 | 77 | 24 | 72 | 41 | 5 | NA | NA | NA | NA | NA | NA | NA |
| 5 | 4 | 2 | 71 | 13 | 21 | 16 | 2 | 6 | 3 | 73 | 14 | 17 | 15 | 1 | 3 | 2 | 78 | 13 | 17 | 15 | 2 |
| 7 | 8 | 4 | 57 | 22 | 32 | 26 | 3 | 11 | 4 | 61 | 25 | 34 | 26 | 4 | *Dropped out* | | | | | | |
| 8 | 6 | 2 | 84 | 29 | 23 | 23 | 2 | 8 | 4 | 96 | 36 | 36 | 37 | 2 | 13 | 7 | 93 | 14 | 28 | 23 | 1.8 |
| *Median* | *7.0* | *2.5* | *84.0* | *22.0* | *41.0* | *26.0* | *2.0* | *11* | *4* | *77* | *24* | *36* | *37* | *2* | *12* | *4* | *211* | *14* | *41* | *34* | *1* |

Supplementary Table 2. *Continued*

| CA  treatment | Week 78 | | | | | | | Week 104 | | | | | | | Week 130 | | | | | | |
| --- | --- | --- | --- | --- | --- | --- | --- | --- | --- | --- | --- | --- | --- | --- | --- | --- | --- | --- | --- | --- | --- |
| Patient # | **TB** | **CB** | **ALP** | **GGT** | **AST** | **ALT** | **AFP** | **TB** | **CB** | **ALP** | **GGT** | **AST** | **ALT** | **AFP** | **TB** | **CB** | **ALP** | **GGT** | **AST** | **ALT** | **AFP** |
| 1 | 13 | 6 | 238 | 12 | 52 | 48 | 2 | 9 | 5 | **298** | 11 | 31 | 29 | 1 | 12 | 5 | 289 | 13 | 33 | 39 | 1 |
| 2^a^ | 8 | 4 | 218 | 9 | 62 | 55 | <1 | 5 | 3 | 179 | 14 | 51 | 58 | <1 | 6 | 3 | 211 | 17 | 43 | 45 | <1 |
| 3^a^ | 6 | 3 | 250 | 25 | **80** | **100** | 2 | 12 | 5 | 279 | 26 | **132** | **139** | 1 | 14 | 6 | 273 | 20 | 67 | 66 | <1 |
| 4 | 11 | 4 | 97 | 38 | 69 | 43 | 4 | 9 | 4 | 85 | 30 | 38 | 30 | 4 | 11 | 4 | 79 | 32 | 45 | 28 | 5 |
| 5 | *Dropped out* | | | | | | | | | | | | | | | | | | | | |
| 7 |  | | | | | | | | | | | | | | | | | | | | |
| 8 | .. | .. | .. | .. | .. | .. | .. | .. | .. | .. | .. | .. | .. | .. | .. | .. | .. | .. | .. | .. | .. |
| *Median* | *9.5* | *4* | *228* | *18.5* | *65.5* | *51.5* | *2* | *9* | *4.5* | *229* | *20* | *44.5* | *44* | *1* | *11.5* | *4.5* | *242* | *18.5* | *44* | *42* | *1* |

Supplementary Table 2. *Continued*

| CA  treatment | Week 156 | | | | | | | Week 182 | | | | | | |
| --- | --- | --- | --- | --- | --- | --- | --- | --- | --- | --- | --- | --- | --- | --- |
| Patient # | **TB** | **CB** | **ALP** | **GGT** | **AST** | **ALT** | **AFP** | **TB** | **CB** | **ALP** | **GGT** | **AST** | **ALT** | **AFP** |
| 1 | 20 | 10 | 286 | 10 | 34 | 21 | 1.5 | 15 | 8 | 205 | 10 | 26 | 19 | 2 |
| 2^a^ | 5 | 3 | 139 | 8 | 40 | 37 | <1 | 5 | 3 | 139 | 9 | 35 | 28 | <1 |
| 3^a^ | 10 | 4 | 230 | 21 | 49 | 54 | 1 | 12 | 7 | 225 | 23 | 56 | 61 | <1 |
| 4 | 11 | 4 | 97 | 38 | 69 | 43 | 4 | 15 | 7 | 71 | 35 | 60 | 34 | 6 |
| 5 |  | | | | | | | | | | | | | |
| 7 |  | | | | | | | | | | | | | |
| 8 | .. | .. | .. | .. | .. | .. | .. | .. | .. | .. | .. | .. | .. | .. |
| *Median* | *10.5* | *4* | *184.5* | *15.5* | *44.5* | *40* | *1.5* | *13.5* | *7* | *172* | *16.5* | *45.5* | *31* | *4* |

^a^ Siblings. *Abbreviations:* CA: cholic acid, TB: total bilirubine, CB: conjugated bilirubin, ALP: alkaline phosphatase, GGT: gamma-glutamyl transferase, AST: aspartate aminotransferase, ALT: alanine aminotransferase, AFP: alpha-fetoprotein *,* NA: data not available. TB and CB are presented in µmol/l; ALP, GGT, AST and ALT are presented in U/L; AFP is presented in µg/L. *Reference range (adults)*: TB: 3-19 µmol/L, CB: <5 µmol/L, ALP: <123 U/L (37°C), GGT: <62 U/L (37°C), AST: 13-38 U/L (37°C), ALT: <34 U/L (37°C), AFP: <7 µg/L. *Reference range (children)*: TB: 4-29 µmol/L, CB: <5 µmol/L, ALP: <455 U/L (37°C), GGT: <36 U/L (37°C), AST: 14-39 U/L (37°C), ALT: <39 U/L (37°C), AFP: <7 µg/L. Note: Significance of bold: value is ≥ 2 x ULN (upper limit of normal). Time point has not yet been reached (..).
