# Supplementary material for: Effectiveness and Safety of Personalized Cholic Acid Treatment in Patients With Bile Acid Synthesis Defects
Source: J Inherit Metab Dis. 2025 Jul 11;48(4):e70062. doi: 10.1002/jimd.70062 (PMC12247693; doi:10.1002/jimd.70062)
Supplement: Supplementary file 3 — Table S3. Coagulation factors and cholesterol tests of AMACR (#1–7) and 3β‐HSD (#8) patients treated with CA. [file JIMD-48-0-s003.docx]

Supplementary Table 3. Coagulation factors and cholesterol tests of AMACR (#1-7) and 3β-HSD (#8) patients treated with CA

| CA  treatment | Week 0 | | | | | Week 6 | | | | | Week 12 | | | | | Week 26 | | | | |
| --- | --- | --- | --- | --- | --- | --- | --- | --- | --- | --- | --- | --- | --- | --- | --- | --- | --- | --- | --- | --- |
| Patient # | **PT** | **aPTT** | **FV** | **FVII** | **Total Cholesterol** | **PT** | **aPTT** | **FV** | **FVII** | **Total Cholesterol** | **PT** | **aPTT** | **FV** | **FVII** | **Total Cholesterol** | **PT** | **aPTT** | **FV** | **FVII** | **Total Cholesterol** |
| 1 | 11.6 | 30 | 93 | 70 | 2.77 | 11.5 | 30 | NA | NA | NA | 11.6 | 30 | NA | NA | NA | 11.4 | 29 | 78 | 68 | 3.06 |
| 2^a^ | 11.3 | 33 | 93 | 75 | 2.37 | 11.4 | 32 | 80 | 66 | 2.86 | 11.0 | 33 | 97 | 87 | 3.06 | NA | NA | NA | NA | NA |
| 3^a^ | 11.9 | 26 | 93 | 50 | 2.13 | 11.8 | 27 | 87 | 52 | 3.05 | 11.3 | 28 | 95 | 78 | 3.29 | NA | NA | NA | NA | NA |
| 4 | 11.4 | 27 | 102 | 65 | 2.95 | 11.6 | 28 | 99 | 62 | 4.43 | 11.5 | 27 | 110 | 79 | 4.68 | 11.6 | 28 | 86 | 72 | 4.46 |
| 5 | 11.9 | 27 | 104 | 46 | 2.95 | 11.9 | 28 | 99 | 53 | 3.95 | 11.9 | 27 | 101 | 48 | 3.69 | 11.4 | 27 | 93 | 57 | 4.09 |
| 7 | 11.4 | 25 | 89 | 52 | 3.63 | 11.3 | 23 | 108 | 58 | 4.01 | 11.2 | 26 | 131 | 62 | 3.46 | 11.5 | 26 | 101 | 54 | 4.13 |
| 8 | 12.1 | 21 | 148 | 39 | 3.09 | 10.9 | 20 | 150 | 75 | 3.87 | 11.1 | 22 | 128 | 59 | 5.74 | 11.4 | 21 | 135 | 53 | 4.50 |
| *Median* | *11.6* | *27* | *93* | *52* | *2.95* | *11.5* | *28* | *99* | *60* | *3.91* | *11.3* | *27* | *105.5* | *70* | *3.575* | *11.45* | *27.5* | *89.5* | *62.5* | *4.11* |

Supplementary Table 3. *Continued*

| CA  treatment | Week 52 | | | | | Week 104 | | | | | Week 156 | | | | |
| --- | --- | --- | --- | --- | --- | --- | --- | --- | --- | --- | --- | --- | --- | --- | --- |
| Patient # | **PT** | **aPTT** | **FV** | **FVII** | **Total Cholesterol** | **PT** | **aPTT** | **FV** | **FVII** | **Total Cholesterol** | **PT** | **aPTT** | **FV** | **FVII** | **Total Cholesterol** |
| 1 | 11.9 | 30 | 75 | 67 | 3.15 | 11.4 | 32 | 80 | 74 | 3.15 | 11.9 | 31 | 72 | 52 | 2.9 |
| 2^a^ | 11.2 | 33 | 131 | 98 | 2.93 | 10.9 | 35 | 79 | 91 | 2.95 | 11.9 | 34 | 73 | 67 | 2.27 |
| 3^a^ | 12.0 | 27 | 92 | 55 | 2.45 | 11.4 | 28 | 82 | 64 | 2.74 | 13.0 | 31 | 77 | 40 | 1.99 |
| 4 | NA | NA | NA | NA | NA | 11.1 | 26 | 106 | 91 | 4.03 | 11.5 | 27 | 97 | 63 | 3.94 |
| 5 | 11.5 | 27 | 88 | 52 | 3.64 | *Dropped out* | | | | | | | | | |
| 7 | *Dropped out* | | | | | | | | | | | | | | |
| 8 | 11.4 | 24 | 146 | 57 | 3.59 | .. | .. | .. | .. | .. | .. | .. | .. | .. | .. |
| *Median* | *11.7* | *28.5* | *90* | *61* | *3.04* | *11.25* | *30* | *81* | *82.5* | *3.05* | *11.9* | *31* | *75* | *57.5* | *2.59* |

^a^ Siblings. *Abbreviations*: CA: cholic acid, PT: prothrombin time, aPTT: activated partial thromboplastin time, FV: Factor-V activity, FVII: Factor-VII activity, NA: data not available. PT and aPPT are presented in seconds, FV and FVII are presented in %, total cholesterol is presented in mmol/L. *Reference values*: PT: 9.7-11.9 seconds, aPPT: 22-29 seconds, FV: 80-140%, FVII: 80-140%, total cholesterol: <5.0 mmol/L. Note: Significance of bold: value is ≥ 2 x ULN (upper limit of normal). Significance of underlined: value is ≤ 2 x ULN. Time point has not yet been reached (..).
