# Supplementary material for: Effectiveness and Safety of Personalized Cholic Acid Treatment in Patients With Bile Acid Synthesis Defects
Source: J Inherit Metab Dis. 2025 Jul 11;48(4):e70062. doi: 10.1002/jimd.70062 (PMC12247693; doi:10.1002/jimd.70062)
Supplement: Supplementary file 4 — Table S4. Plasma tests of long chain fatty acids, phytanic acid, pristanic acid, and fat‐soluble vitamins of AMACR (#1–7) and 3β‐HSD (#8) patients treated with CA. [file JIMD-48-0-s002.docx]

Supplementary Table 4. Plasma tests of long chain fatty acids, phytanic acid and pristanic acid and fat soluble vitamins of AMACR of AMACR (#1-7) and 3β-HSD (#8) patients treated with CA

| CA  treatment | Week 0 | | | | | | | | Week 12 | | | | | | | |
| --- | --- | --- | --- | --- | --- | --- | --- | --- | --- | --- | --- | --- | --- | --- | --- | --- |
| Patient # | **C22:0** | **C24:0** | **C26:0-**  **LysoPC** | **C29** | **Pristanic**  **acid** | **Vitamin A** | **Vitamin D** | **Vitamin E** | **C22:0** | **C24:0** | **C26:0-**  **LysoPC** | **C29** | **Pristanic**  **acid** | **Vitamin A** | **Vitamin D** | **Vitamin E** |
| 1 | 55 | 46 | NA | **0.7** | **17.1** | 1.3 | 79.0 | 17.9 | NA | NA | 11 | **0.2*** | NA | NA | NA | NA |
| 2^a^ | 72 | 53 | NA | **0.3** | **10.9** | 1.0 | 15.0 | 26.0 | 63 | 50 | NA | 0.0***** | **10.7** | 1.0 | 50.0 | 19.4 |
| 3^a^ | 57 | 43 | NA | **0.2** | **7.1** | 0.9 | 28.0 | 17.8 | 60 | 49 | NA | NA | **9.4** | 1.2 | 56.0 | 27.6 |
| 4 | 54 | 41 | 21 | **0.6** | **101.0** | 1.6 | 101.0 | 30.1 | 61 | 49 | NA | 0.0***** | **86.3** | 1.4 | 91.0 | 37.1 |
| 5 | 57 | 38 | 19 | **0.5** | **27.2** | 1.1 | 48.0 | 19.0 | 60 | 42 | 23 | 0.0***** | **38.5** | 0.9 | 66.0 | 22.9 |
| 7 | 62 | 46 | NA | NA | **98.0** | 2.0 | 82.0 | 26.1 | 56 | 40 | NA | **0.3** | **69.2** | 1.8 | 67.0 | 22.1 |
| 8 | 23 | 19 | NA | 0.0 | NA | 0.8 | 39.0 | 20.2 | 90 | 59 # | NA | 0.0 | NA | 2.4 | 53.0 | 42.4 |
| *Median* | *57* | *43* | *20* | *0.4* | *22.2* | *1.1* | *48.0* | *20.2* | *61* | *49* | *17* | *0.0* | *38.5* | *1.3* | *61.0* | *25.3* |

Supplementary Table 4. *Continued*

| CA  treatment | Week 26 | | | | | | | | Week 52 | | | | | | | |
| --- | --- | --- | --- | --- | --- | --- | --- | --- | --- | --- | --- | --- | --- | --- | --- | --- |
| Patient # | **C22:0** | **C24:0** | **C26:0-**  **LysoPC** | **C29** | **Pristanic**  **acid** | **Vitamin A** | **Vitamin D** | **Vitamin E** | **C22:0** | **C24:0** | **C26:0-**  **LysoPC** | **C29** | **Pristanic**  **acid** | **Vitamin A** | **Vitamin D** | **Vitamin E** |
| 1 | 54 | 47 | NA | 0.0* | **10.6** | 1.0 | 73.0 | 18.7 | NA | NA | 9 | 0.0* | **16.2** | 1.0 | 70.0 | 18.6 |
| 2^a^ | 53 | 47 | 11 | 0.0***** | **8.6** | NA | NA | NA | NA | NA | NA | **0.1*** | **8.5** | 1.1 | 31.0 | 20.3 |
| 3^a^ | 49 | 48 | 14 | 0.0***** | **11.1** | NA | NA | NA | NA | NA | NA | 0.0***** | **12.9** | 1.2 | 33.0 | 19.4 |
| 4 | 65 | 44 | NA | 0.0***** | **101** | 1.5 | 96.0 | 29.6 | NA | NA | NA | NA | NA | NA | NA | NA |
| 5 | 69 | 47 | NA | NA | **33.4** | 1.3 | 63.0 | 28.6 | 54 | 40 | NA | 0.0***** | **36.0** | 0.9 | 49.0 | 21.2 |
| 7 | 65 | 52 | NA | 0.0 | **107.3** | 1.7 | 80.0 | 25.7 | *Dropped out* | | | | | | | |
| 8 | NA | NA | NA | NA | NA | 2.4 | 46.0 | 42.9 | 6.4* | 15.8 | 29 | NA | NA | 1.8 | 36 | 27.5 |
| *Median* | *60* | *47* | *13* | *0.0* | *22.3* | *1.4* | *76.5* | *27.2* | *54* | *40* | *9* | *0.0* | *14.6* | *1.1* | *41.0* | *19.9* |

Supplementary Table 4. *Continued*

| CA  treatment | Week 78 | | | | | | | | Week 104 | | | | | | | |
| --- | --- | --- | --- | --- | --- | --- | --- | --- | --- | --- | --- | --- | --- | --- | --- | --- |
| Patient # | **C22:0** | **C24:0** | **C26:0-**  **LysoPC** | **C29** | **Pristanic**  **acid** | **Vitamin A** | **Vitamin D** | **Vitamin E** | **C22:0** | **C24:0** | **C26:0-**  **LysoPC** | **C29** | **Pristanic**  **acid** | **Vitamin A** | **Vitamin D** | **Vitamin E** |
| 1 | NA | NA |  | 0.0* |  | NA | NA | NA | 56 | 46 | 30 | **0.1*** | **15.9** | 1.4 | 68.0 | 20.8 |
| 2^a^ | NA | NA | 14 | **0.1*** | **16.3** | NA | NA | NA | 72 | 59 | 22 | **0.1*** | **11.8** | 1.0 | 23.0 | 27.1 |
| 3^a^ | NA | NA | 17 | 0.0***** | **14.7** # | NA | NA | NA | 70 | 58 | 26 | **0.1*** | **11.5** | 1.5 | 30.0 | 29.7 |
| 4 | NA | NA | NA | NA | NA | NA | NA | NA | 58 | 50 | 30 | **0.1*** | **103.8** | 1.5 | 100.0 | 27.8 |
| 5 | *Dropped out* | | | | | | | | | | | | | | | |
| 7 |  | | | | | | | | | | | | | | | |
| 8 | .. | .. | .. | .. | .. | .. | .. | .. | .. | .. | .. | .. | .. | .. | .. | .. |
| *Median* | *NA* | *NA* | *16* | *0.0* | *15.5* | NA | NA | NA | *64* | *54* | *28* | *0.1* | *13.9* | *1.5* | *49.0* | *27.5* |

Supplementary Table 4. *Continued*

| CA  treatment | Week 130 | | | | | | | | Week 156 | | | | | | | |
| --- | --- | --- | --- | --- | --- | --- | --- | --- | --- | --- | --- | --- | --- | --- | --- | --- |
| Patient # | C22:0 | **C24:0** | **C26:0-**  **LysoPC** | **C29** | **Pristanic**  **acid** | **Vitamin A** | **Vitamin D** | **Vitamin E** | **C22:0** | **C24:0** | **C26:0-**  **LysoPC** | **C29** | **Pristanic**  **acid** | **Vitamin A** | **Vitamin D** | **Vitamin E** |
| 1 | NA | NA | 25 | NA | **14.8** | NA | NA | NA | 56 | 48 | 22 | **0.1*** | **11.1** | 1.6 | 45.0 | 18.2 |
| 2^a^ | NA | NA | 17 | **0.2** | **9.2** | NA | NA | NA | 43 | 35 | 15 | 0.0* | **4.9** | 0.9 | 16.0 | 13.1 |
| 3^a^ | NA | NA | 12 | **0.1*** | **12.9** | NA | NA | NA | 20* | 24 | 12 | 0.0***** | **4.9** | 1.0 | 30.0 | 12.8 |
| 4 | NA | NA | 28 | NA | **92.5** | NA | NA | NA | 49 | 31 | 24 | NA | **40.5*** | 2.2 | 103.0 | 29.0 |
| 5 |  | | | | | | | | | | | | | | | |
| 7 |  | | | | | | | | | | | | | | | |
| 8 | .. | .. | .. | .. | .. | .. | .. | .. | .. | .. | .. | .. | .. | .. | .. | .. |
| *Median* | *NA* | *NA* | *21.0* | *0.2* | *13.9* | NA | NA | NA | *46* | *33* | *19* | *0.0* | *8.0* | *1.3* | *37.5* | *15.7* |

Supplementary Table 4. *Continued*

| CA  treatment | Week 182 | | | | |  |  |  |
| --- | --- | --- | --- | --- | --- | --- | --- | --- |
| Patient # | **C22:0** | **C24:0** | **C26:0-**  **LysoPC** | **C29** | **Pristanic**  **acid** | **Vitamin A** | **Vitamin D** | **Vitamin E** |
| 1 | NA | NA | 23 | **0.1** | **6.05** | NA | NA | NA |
| 2^a^ | NA | NA | 20 | 0.0 | **5.70** | NA | NA | NA |
| 3^a^ | NA | NA | 14 | **0.1** | **6.21** | NA | NA | NA |
| 4 | NA | NA | 29 | **0.1** | **59.58** | NA | NA | NA |
| 5 |  | | | | | | | |
| 7 |  | | | | | | | |
| 8 | .. | .. | .. | .. | .. | .. | .. | .. |
| *Median* | *NA* | *NA* | *21.5* | *0.1* | *6.13* | NA | NA | NA |

^a^ Siblings. *Abbreviations:* CA: cholic acid, C22:0: behenic acid, C24:0: Lignoceric acid, C26:0-LysoPC: C26:0 lysophosphatidylcholine, C29: C29-galdicarboxylic acid, NA: data not available. Plasma fatty acids, Vitamin A and E are presented in µmol/l, vitamin D is presented in nmol/L. R*eference range*: C22:0: 42 – 81 µmol/L, C24:0: 28 – 57 µmol/L, C26:0-LysoPC: 29 – 88 nmol/L, C29: 0-0.001 µmol/L, pristanic acid: 0 – 1.6 µmol/L, vitamin A: 1.2-2.7 µmol/L, vitamin D: >50 nmol/L, vitamin E: 15-35 µmol/L. *Reference values (children)*: vitamin A: 0.7-2.9 µmol/L, vitamin D: >50 nmol/L, vitamin E: 20-42 µmol/L. *Reference values (children)*: vitamin A: 0.7-2.9 µmol/L, vitamin D: >50 nmol/L, vitamin E: 20-42 µmol/L. Note: Significance of bold: value is ≥ 2 x ULN (upper limit of normal). Significance underlined: value is ≤ 0.5 x LLN (lower limit of normal). Phytanic acid plasma levels were normal (within reference range 0.49-9.88 µmol/L) for all patients at all time points (data not shown). *Plasma value is ≤ 0.5 x baseline value. # Plasma value is ≥ 2 x baseline value. Time point has not yet been reached (..).
